# Supplementary material for: Effects of Once-Weekly Semaglutide on Cardiovascular Risk Factors and Metabolic Dysfunction-Associated Steatotic Liver Disease in Japanese Patients with Type 2 Diabetes: A Retrospective Longitudinal Study Based on Real-World Data
Source: Biomedicines. 2024 May 2;12(5):1001. doi: 10.3390/biomedicines12051001 (PMC11118092; doi:10.3390/biomedicines12051001)
Supplement: Supplementary file 1 [file biomedicines-12-01001-s001.zip › biomedicines-2934221-supplementary.pdf]

Table S1. The changes in metabolic parameters during the semaglutide treatment in patients who were GLP-1RA naïve or given semaglutide after being switched from other GLP-1RAs.

(a) The changes in metabolic parameters 3 months after the initiation of semaglutide treatment.

	Group A GLP-1RA naïve (n=36)				Group B Switch from other GLP-1RAs (n=39)			
	n	Baseline	3 months	*p*	n	Baseline	3 months	*p*
Body weight (kg)	31	90.6 ± 17.3	88.4 ± 16.6	0.019	33	78.9 ± 10.9	78.2 ± 11.5	0.086
BMI (kg/m^2)	31	33.7 ± 5.2	32.9 ± 5.2	0.020	33	29.4 ± 4.2	29.1 ± 4.1	0.051
Systolic blood pressure (mmHg)	32	135 ± 15	130 ± 14	0.162	33	132 ± 12	129 ± 14	0.292
Diastolic blood pressure (mmHg)	32	80 ± 10	81 ± 12	0.557	33	78 ± 13	77 ± 12	0.637
Plasma glucose (mg/dL)	34	181 ± 65	155 ± 47	< 0.001	39	166 ± 46	171 ± 50	0.635
HbA1c (%)	34	8.3 ± 1.4	7.4 ± 1.0	< 0.001	39	8.1 ± 1.2	7.6 ± 0.9	0.001
Alb (g/dL)	33	4.10 ± 0.53	4.16 ± 0.41	0.243	35	4.14 ± 0.57	4.21 ± 0.46	0.228
AST (IU/L)	36	40 ± 26	28 ± 12	< 0.001	39	35 ± 33	31 ± 25	0.404
ALT (IU/L)	36	54 ± 38	38 ± 23	< 0.001	39	43 ± 36	40 ± 36	0.244
gGTP (IU/L)	34	66 ± 61	51 ± 44	< 0.001	38	57 ± 60	52 ± 71	0.152
TC (mg/dL)	33	190 ± 45	166 ± 28	< 0.001	36	167 ± 29	166 ± 33	0.489
HDL-C (mg/dL)	33	47 ± 11	48 ± 11	0.250	38	45 ± 10	45 ± 11	0.349
LDL-C (mg/dL)	27	101 ± 29	87 ± 25	0.004	35	90 ± 25	89 ± 24	0.823
TG (mg/dL)	32	222 ± 136	178 ± 85	0.014	38	227 ± 159	227 ± 164	0.520
TG / HDL-C	33	5.1 ± 3.4	3.9 ± 2.2	0.013	38	5.7 ± 4.9	6.1 ± 6.5	0.348
Non-HDL-C (mg/dL)	34	140 ± 44	118 ± 27	< 0.001	36	121 ± 29	121 ± 34	0.421
Creatinine (mg/dL)	36	0.83 ± 0.34	0.88 ± 0.70	0.655	39	0.78 ± 0.31	0.79 ± 0.29	0.170
eGFR (mL/min/1.73 m^2)	36	74 ± 22	76 ± 25	0.179	39	84 ± 36	81 ± 33	0.142
Uric acid (mg/dL)	30	5.6 ± 1.6	5.3 ± 1.8	0.150	36	5.9 ± 1.5	5.8 ± 1.5	0.640
Hemoglobin (g/dL)	36	14.5 ± 1.7	14.4 ± 1.4	0.494	39	14.6 ± 1.8	14.7 ± 1.7	0.514
Platelet (× 10^4/μL)	36	24.8 ± 7.6	25.3 ± 7.8	0.254	39	23.4 ± 6.7	24.2 ± 8.1	0.207
UACR (mg/g Cre)	15	210 ± 402	145 ± 266	0.307	23	131 ± 186	94 ± 185	0.236

(b) The changes in metabolic parameters 6 months after the initiation of semaglutide treatment.

	GLP-1RA naïve (n=29)				Switch from other GLP-1RAs (n=25)			
	n	Baseline	6 months	*p*	n	Baseline	6 months	*p*
Body weight (kg)	26	89.4 ± 18.3	86.8 ± 18.1	0.035	24	78.8 ± 11.0	78.0 ± 11.1	0.131
BMI (kg/m^2)	26	33.1 ± 5.3	32.1 ± 5.5	0.031	24	29.6 ± 4.6	29.3 ± 4.7	0.101
Systolic blood pressure (mmHg)	27	134 ± 14	133 ± 19	0.849	24	129 ± 12	133 ± 14	0.224
Diastolic blood pressure (mmHg)	27	79 ± 10	80 ± 11	0.756	24	76 ± 9	79 ± 10	0.298
Plasma glucose (mg/dL)	28	189 ± 67	150 ± 44	0.001	25	166 ± 45	177 ± 50	0.387
HbA1c (%)	28	8.5 ± 1.5	7.3 ± 1.0	< 0.001	25	8.2 ± 1.3	7.7 ± 0.9	0.027
Alb (g/dL)	28	4.12 ± 0.38	4.12 ± 0.38	1.000	20	4.05 ± 0.67	4.20 ± 0.50	0.187
AST (IU/L)	29	42 ± 27	30 ± 13	0.003	25	33 ± 31	32 ± 30	0.692

ALT (IU/L)	29	56 ± 40	38 ± 19	0.002	25	42 ± 31	41 ± 36	0.202
GGTP (IU/L)	27	72 ± 67	51 ± 47	< 0.001	24	49 ± 51	52 ± 79	0.537
TC (mg/dL)	27	192 ± 48	170 ± 28	0.038	23	164 ± 26	164 ± 30	0.983
HDL-C (mg/dL)	27	48 ± 11	50 ± 12	0.314	25	44 ± 10	45 ± 12	0774
LDL-C (mg/dL)	22	101 ± 30	91 ± 20	0.226	24	91 ± 26	86 ± 20	0.229
TG (mg/dL)	27	234 ± 149	175 ± 96	0.005	25	218 ± 127	256 ± 253	0.732
TG / HDL-C	27	5.3 ± 3.6	3.9 ± 2.6	0.013	25	5.7 ± 4.6	7.3 ± 9.6	0.882
Non-HDL-C (mg/dL)	26	142 ± 47	121 ± 29	0.054	23	119 ± 28	119 ± 34	0.975
Creatinine (mg/dL)	29	0.84 ± 0.37	0.81 ± 0.40	0.208	25	0.80 ± 0.33	0.81 ± 0.35	0.762
eGFR (mL/min/1.73 m^2)	29	74 ± 24	78 ± 24	0.064	25	85 ± 41	85 ± 41	0.929
Uric acid (mg/dL)	24	5.8 ± 176	5.3 ± 2.4	0.103	23	6.0 ± 1.6	5.7 ± 1.2	0.128
Hemoglobin (g/dL)	29	14.6 ± 1.5	14.3 ± 1.1	0.146	25	14.3 ± 2.0	14.6 ± 1.7	0.246
Platelet (× 10^4/μL)	29	23.6 ± 6.9	24.4 ± 6.9	0.138	25	23.3 ± 6.6	23.4 ± 6.3	0.866
UACR (mg/g Cre)	13	216 ± 445	166 ± 300	0.753	15	97 ± 175	99 ± 163	0.280

Values show mean ± SD. ALT, alanine aminotransferase; AST, aspartate aminotransferase; BMI, body mass index; eGFR, estimated glomerular filtration rate; GGTP, gamma-glutamyl transferase; HbA1c, hemoglobin A1c; HDL-C, high-density lipoprotein cholesterol; LDL-C, low-density lipoprotein cholesterol; TC, total cholesterol; TG, triglyceride; UACR, albumin-to-creatinine ratio.
